# Zooming in on the Glycome: Visualizing Glycans at the Nanoscale Level through Expansion Microscopy

**DOI:** 10.1021/acscentsci.4c01836

**Published:** 2024-11-25

**Authors:** Joshua M. Kofsky, Chantelle J. Capicciotti

**Affiliations:** † Department of Chemistry, Department of Biomedical and Molecular Sciences, Department of Surgery, Queen’s University, Kingston, Ontario K7L 3N6, Canada; ‡ Department of Chemistry, Queen’s University, Kingston, Ontario K7L 3N6, Canada

Glycans are complex carbohydrate structures that decorate many proteins and lipids within and on the surface of every cell. They are critical for proper cellular, tissue, and organ function, and dysregulation of glycosylation is implicated in many diseases. Despite their abundance and necessity, two main questions have limited our understanding of glycan-mediated processes: where are specific glycans located within multicellular systems and what are the specific glycan structures responsible for the myriad of biological functions that glycans have? In this issue of *ACS Central Science,* a team co-led by Christopher Alabi and Matthew Paszek developed a new expansion microscopy (ExM) technique to aid in addressing these questions, enabling researchers to “zoom in” on glycans to image and map them within cells, tissues, and whole nematode organisms.^1^ By coupling metabolic incorporation of azide-functionalized glycans with expansion microscopy, this work provides an expanded, nanoscale view of the glycome.

**Figure 1:**
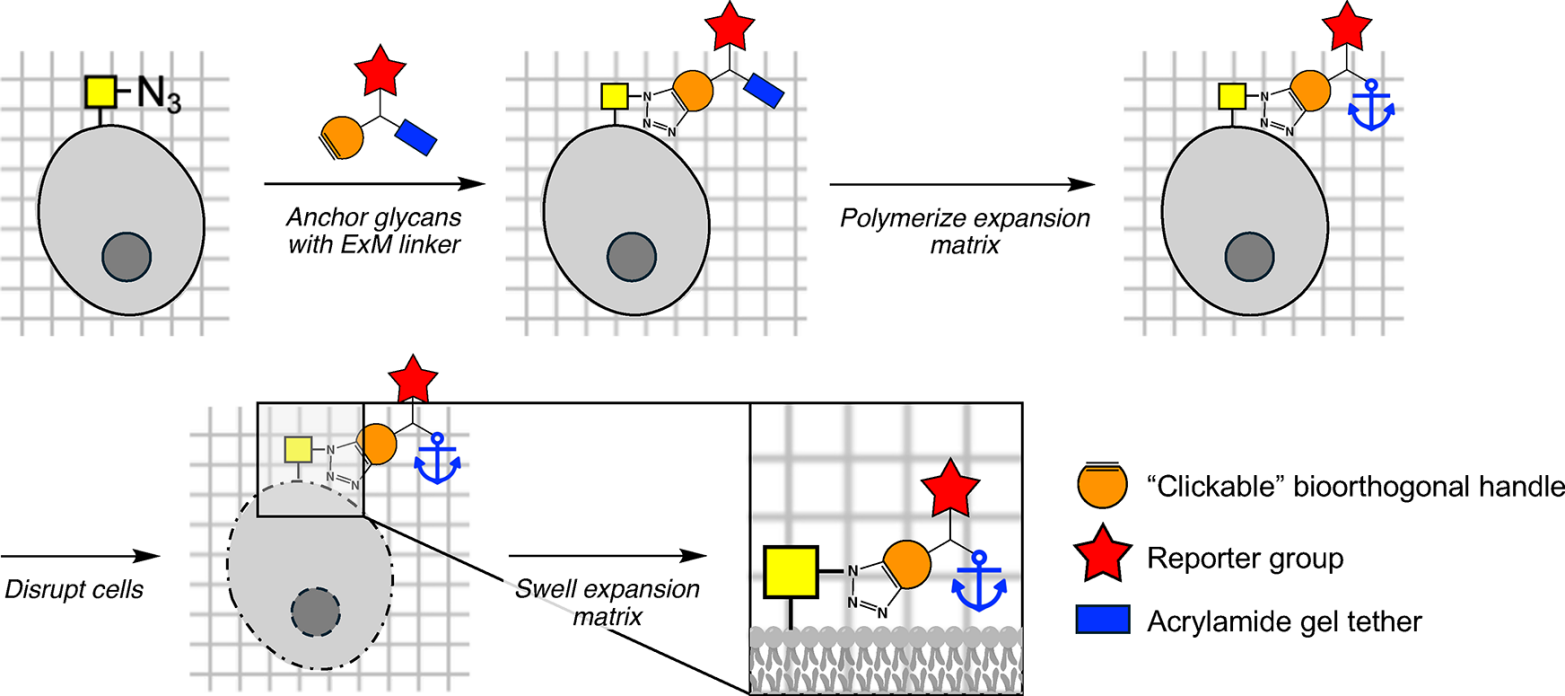
Expansion microscopy “zooms in” on analytes like glycans and proteins at the nanoscale.

ExM is a sample preparation technique that uses swellable polymer matrices to expand samples with minimal spatial distortion, providing improved spatial resolution of finer biological structures using diffraction-limited optical microscopes. This technique greatly reduces optical strength requirements, enabling precise nanoscale imaging on conventional microscopes, thereby broadening the scope of analytes that can be imaged with a robust, cost-effective technique at resolutions often reserved for electron microscopy.^2^ In ExM, analytes of interest (e.g., biomolecules or their fluorescent probes) are chemically tethered to an expansion matrix, and cells are disrupted to allow the sample to expand isotropically during matrix swelling (Figure 1); during this process, analytes not anchored in the expansion matrix may be lost. While protocols have been developed for ExM imaging of proteins, DNA, and RNA, direct enhanced resolution imaging of other biomolecules like glycans remains challenging, notably in multicellular systems and organisms which are hampered by the tissue penetrance of ExM probes.

**Figure 2:**
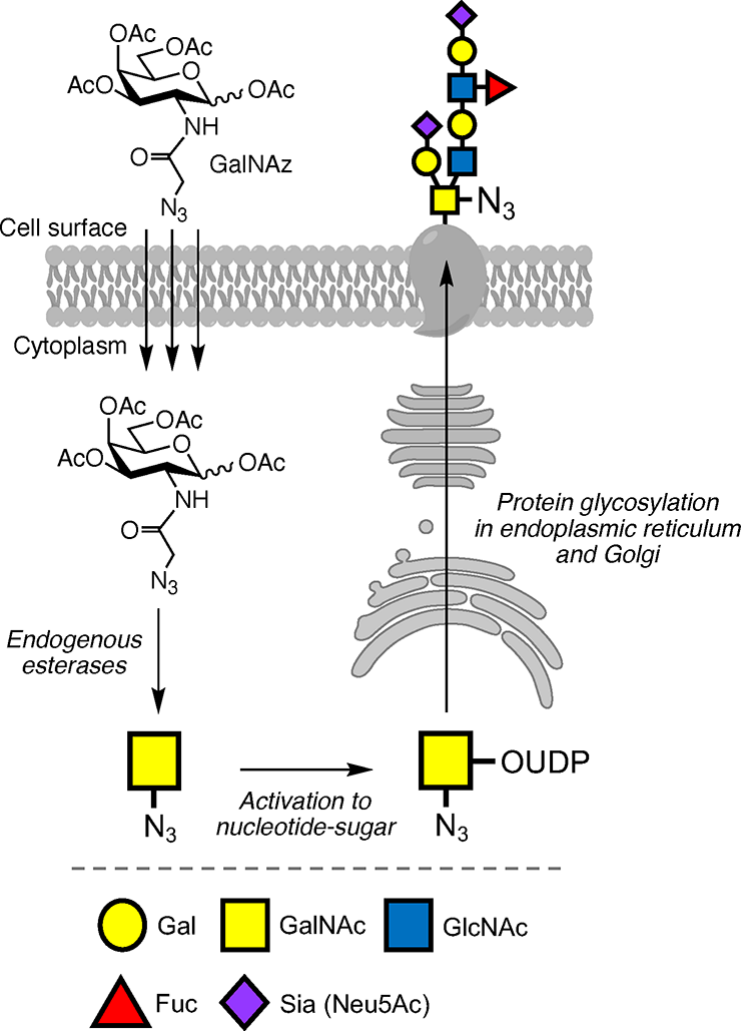
Metabolic incorporation of azide-containing GalNAz probes through endogenous glycosylation machinery to incorporate probes in O-glycan structures. Monosaccharide symbols follow the SNFG (Symbol Nomenclature for Glycans) system.^9^

The defined molecular sequences of DNA, RNA, and proteins has allowed for their specific targeting by fluorescent imaging probes, or genetic encoding of fluorescent tags, which has facilitated ExM of these biomolecules. In contrast, glycan sequences are not templated in the genome; instead, their biosynthesis is regulated by glycosyltransferase and hydrolase enzymes with overlapping activities and specificities. This makes common genetic manipulation techniques not ideal for studying glycans and glycoconjugates. One of the most versatile and important tools to label and visualize glycans is metabolic oligosaccharide engineering (MOE), where monosaccharides bearing chemical reporters can be metabolically incorporated into cellular glycans.^3^ In MOE, protected monosaccharide probes diffuse across cell membranes, and after protecting group removal by endogenous enzymes, monosaccharides with chemical reporters (e.g., azides) are revealed that are incorporated into glycans by native cellular glycosylation machinery (Figure 2). Detection of azide-labeled glycans is then achieved through bioorthogonal “click” reactions with an alkyne-fluorophore.

Kuo, Colville, Sorkin et al. used MOE with two azide-containing monosaccharides: a *N-*(azidoacetyl)mannosamine derivative (ManNAz), which is metabolized into azidoacetyl sialic acid (SiaNAz) terminating glycans, and a *N*-(azidoacetyl)galactosamine derivative (GalNAz), which is metabolically incorporated into mucin-type O-glycans (Figure 2).^1^ The authors report an elegant and facile design of a library of trifunctional ExM linkers that were used to image metabolically labeled glycans through ExM by directly anchoring glycan probes to the expansion matrix. Bridged off an oligothioetheramide core (oligoTEA), their ExM linkers comprised: 1) a bioorthogonal click handle (alkyne or tetrazine) to conjugate to metabolically labeled glycans; 2) a reporter molecule (fluorescent dye or biotin) for visualization of the glycans; and 3) a gel tether for anchoring within the swellable expansion matrix.

The “clickable” ExM linkers were first used to image cultured breast cancer cells engineered with SiaNAz-terminating glycans through MOE. After “clicking” the ExM linker to glycans, anchoring to the gel matrix, and subsequent matrix expansion, SiaNAz-bearing glycans present on fine ultrastructures of the cell membrane were resolved at the nanoscale, including migrasomes, microvilli, and membrane blebs that are correlated with aggressive and invasive breast cancers. Importantly, the method is compatible with standard protein ExM protocols and is comparable to scanning electron microscopy, as demonstrated by dual visualization of glycans and their membrane-bound protein using a Mucin-1 glycoprotein as a proof-of-concept. The ability to concurrently image the glycans and protein core of a heavily O-glycosylated glycoprotein is significant. Given that MOE with ManNAz broadly incorporates SiaNAz into a variety of glycan classes and glycoproteins (e.g., N/O-glycans and glycolipids),^4^ this technique holds tremendous promise for multifunctional imaging of other important disease-associated glycans/glycoproteins. Notably, off-target labeling from metabolic incorporation of the azido-sugars used in this work has been well documented.^3^ Applying recent advances in the glycobiology field to selectively label specific glycan subclasses, such as designing selective metabolic probes,^3^ “bump-and-hole” glycosyltransferase engineering,^5^ and selective exo-enzymatic glycan editing,^6^ will vastly improve the precision of ExM-enabled glycan and glycoprotein mapping.

Glycan labeling and ExM imaging was also applied to intact, newly hatched *C. elegans* larvae. While previous work demonstrated that nematodes can metabolically incorporate GalNAz into their O-glycans, labeling was limited to tissues exposed to the labeling solution, with limited resolution of internal features encased in *C. elegans*’ hard cuticle.^7^ By permeabilizing the cuticle with repeated freeze–thaw cycles or mild detergent treatment, the authors revealed O-glycosylation patterns of solution-exposed and cuticle-encased tissues in live and fixed *C. elegans* with stunning resolution. O-Glycosylation of nanoanatomical substructures, such as cuticle folds and metastomal flaps within the buccal cavity, was observed, along with unexpected differential O-glycan patterns and “hotspots” on cuticle furrows that may be lost during sample preparation for standard TEM imaging. This work is a major advancement for mapping glycosylation at the nanoscale in live and fixed organisms without the need for specialized TEM imaging techniques, vastly improving the accessibility of these imaging modalities to a broad range of researchers.

The enlightening ExM strategy reported in this work has expanded the frontier of what is possible for resolving glycans at the nanoscale by anchoring glycans labeled with azides through MOE to an expansion matrix using trifunctional ExM linkers. While this work primarily brings the glycome into focus, the generality of the bioorthogonally functionalized ExM linkers makes this technique useful for researchers spanning many disciplines. Advances in introducing bioorthogonal moieties into other biomolecules like proteins, lipids, and nucleic acids will widen the scope of analytes that can be imaged with these ExM linkers.^8^ Integrating bioorthogonal labels on multiple classes of biomolecules in parallel will enable high-resolution, multiplexed imaging on human tissues and whole complex organisms. It will expand the breadth of possibilities for ExM imaging to ultimately unveil how the complex network of biomolecules interacts to regulate health and disease.
